# A case of biopsy‐proven acute interstitial nephritis following atezolizumab‐bevacizumab treatment of advanced unresectable hepatocellular carcinoma

**DOI:** 10.1002/cnr2.2110

**Published:** 2024-07-25

**Authors:** Reema Patel, Omar Elghawy, Amanda Gibbs, Srishti Gupta, Varinder Kaur

**Affiliations:** ^1^ Department of Internal Medicine, Division of Hematology‐Oncology University of Virginia Charlottesville Virginia USA; ^2^ Department of General Internal Medicine University of Pennsylvania Philadelphia Pennsylvania USA; ^3^ Department of Pathology University of Virginia Charlottesville Virginia USA; ^4^ Department of Hematopathology University of California San Francisco California USA

**Keywords:** atezolizumab, hepatocellular carcinoma, immune related adverse event, immunotherapy, oncology

## Abstract

**Background:**

The advent of immune checkpoint inhibitors (ICIs) represented a significant breakthrough in cancer therapy. Recently, the combined use of atezolizumab and bevacizumab was approved as first‐line treatment for unresectable hepatocellular carcinoma (HCC). Exposure to a novel and diverse spectrum of immune‐related adverse events (irAEs) has increased with the growing utilization of ICIs, however, a comprehensive understanding surrounding newer agents is still lacking. The incidence of kidney toxicities is rare but rising, often underreported due to the lack of confirmatory biopsies. Here, we present a rare case of biopsy‐proven acute interstitial nephritis (AIN) following atezolizumab‐bevacizumab treatment of advanced unresectable HCC.

**Case:**

An 84‐year‐old male with T4N0M0 hepatocellular carcinoma was admitted after cycle 5 of atezolizumab due to decreased urine output and dysuria with a serum creatine of 4.7 mg/dL compared to a baseline of 1.3 mg/dL. To confirm the diagnosis of possible intrinsic renal injury, an ultrasound‐guided non‐focal biopsy of the left kidney was performed, revealing AIN. Potential exacerbatory medications, such as proton‐pump inhibitors, were discontinued. The patient was discharged on oral steroids with improvement in serum creatinine. Before completing the steroid taper, the patient developed pneumocystis pneumonia and eventually transitioned to hospice care.

**Conclusion:**

This case highlights the valuable role renal biopsy can play in accurately capturing irAEs and guiding appropriate management in the setting of ICI‐induced AKI. It also exemplifies important considerations for steroid treatment of irAEs in the setting of comorbidities, such as diabetes.

## 1 INTRODUCTION

Advancements over the past decade have brought novel immunotherapies to the forefront of cancer treatment. Immune checkpoint inhibitors (ICIs), such as anti‐programmed death‐1 (PD1), anti‐programmed death ligand‐1 (PD‐L1), and anti‐cytotoxic T‐lymphocyte‐associated protein 4 inhibitors (CTLA4), have been successfully utilized to treat various cancers including metastatic melanoma, renal cell carcinoma (RCC), and non‐small cell lung cancer. ICIs promote T cell activation and the antitumor immune response, ultimately leading to tumor cell death.^1^ Unfortunately, these drugs have also been associated with T‐cell activation against native tissues, leading to immune‐related adverse effects (irAEs) such as colitis, pneumonitis, hepatitis, and endocrinopathies.^2^

Renal pathologies account for a very small percentage of irAEs, with a reported 2%–5% of patients on PD‐1 inhibitors or combination ICI therapy developing acute kidney injury (AKI).^3^ AKI is pre‐renal in the majority of cases, usually secondary to profound volume depletion or medication use (i.e., non‐steroidal anti‐inflammatory drugs (NSAIDs), ACE inhibitors, diuretics). Once these causes have been excluded, other etiologies must be considered. Previous studies describe acute interstitial nephritis (AIN) as the predominant etiology for intrarenal AKIs in patients receiving ICIs—specifically nivolumab, pembrolizumab, and ipilimumab—for melanoma, urothelial cancer, and lung adenocarcinoma.^3^, ^4^, ^5^ Furthermore, AIN is likely underdiagnosed as many patients do not undergo kidney biopsy to confirm intrinsic pathology.^6^

As ICIs continue to be investigated in clinical trials for approval in additional cancer types, it is crucial to understand the full extent of possible side effects to guide clinical decision‐making and patient management. The combined use of atezolizumab, a PD‐L1 inhibitor, and bevacizumab, a vascular endothelial growth factor (VEGF) inhibitor, was recently approved in 2020 as the first‐line treatment for unresectable hepatocellular carcinoma (HCC) in more than 70 countries following clinically significant data from the IMbrave150 trial.^7^, ^8^ Safety and efficacy testing results found proteinuria, hypertension, and elevated AST to be the most frequent treatment‐related adverse events; less common effects included pneumonia, liver injury, and gastrointestinal hemorrhage.^8^

The incidence of kidney toxicity is rising with increased usage of ICIs, but less remains known about newer drugs like atezolizumab and bevacizumab.^6^ Few cases of AIN in the setting of urothelial carcinoma, in addition to rare instances of mesangial proliferative glomerulonephritis and membranous nephropathy, have been reported.^9^, ^10^, ^11^ The literature on renal irAEs remains sparse, especially surrounding HCC for which atezolizumab‐bevacizumab has only been recently approved. To our knowledge, we present the first reported case of biopsy‐proven acute interstitial nephritis (AIN) following atezolizumab‐bevacizumab treatment of advanced unresectable HCC.

## 2 CASE

**FIGURE 1:**
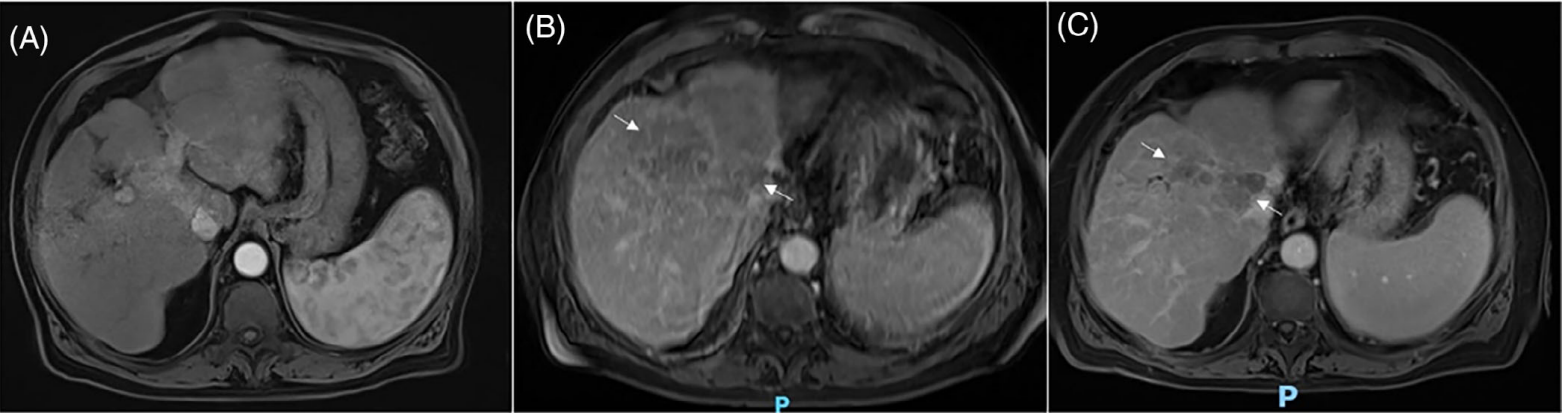
Abdominal MRI. (A) A mass measuring 6.9 × 3.7 cm involving segments 8 and 4A near the IVC with extension into the left and right portal veins can be seen (*arrows*). (B) MRI two months later demonstrated significant tumor growth to 10.0 × 5.8 cm. (C) Follow‐up MRI after four cycles of treatment showed partial tumor response with a reduction to 8.6 × 3.5 cm. There remains a persistent thrombus within the right hepatic veins with some extension into the suprahepatic IVC, overall decreased from prior scans.

An 84‐year‐old male was diagnosed in March of 2022 with moderately differentiated stage IIIB HCC (T4N0M0) via biopsy staining positive for hepatocyte‐specific antigen and negative for cytokeratin 7 and 20. He was initially found to have hypoechoic liver lesions during outpatient surveillance ultrasound imaging with subsequent MRI revealing a 6.9 × 3.7 cm mass involving segments 8 and segment 4A near the IVC with extension into the left and right portal veins, categorized as LIRADS 5 (Figure 1A). Following biopsy results, he was referred to the University of Virginia Cancer Center for further care.

His medical history included cryptogenic cirrhosis, well‐controlled type 2 diabetes mellitus, stage 3 chronic kidney disease, gastroesophageal reflux disease, and hypertension. His home medications were metformin, glimepiride, lisinopril, and omeprazole. Baseline hepatic function measures were only significant for mildly elevated liver enzymes (aspartate transaminase 63 U/L; alanine transaminase 63 U/L). After discussion of his case at tumor board, the decision was made to pursue stereotactic body radiation therapy followed by combined atezolizumab (1200 mg) and bevacizumab (15 mg/kg) every three weeks. He completed five treatments of radiation, receiving a total dose of 50 Gy. After one cycle of combined immunotherapy, he was transitioned to atezolizumab alone due to symptoms of fatigue, weakness, anorexia, and dizziness. Follow‐up MRI of the liver after four cycles demonstrated partial response of the tumor with reduced venous thrombi extent and enhancement (Figure 1B).

**FIGURE 2:**
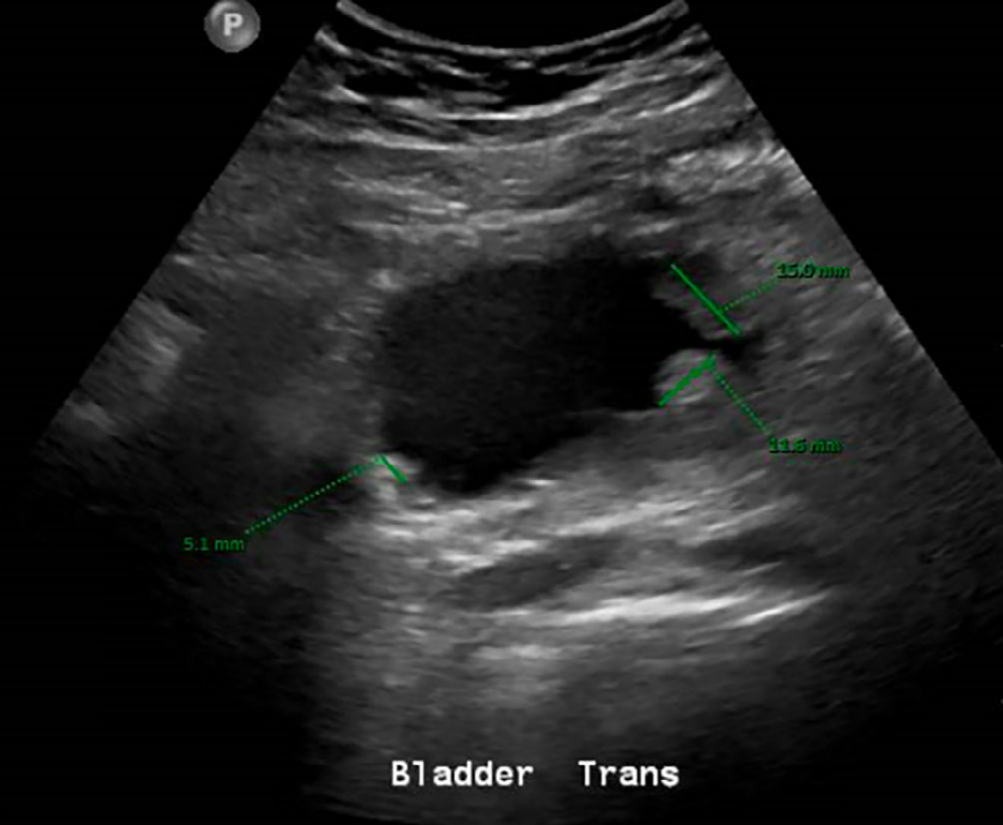
Renal ultrasound. Three urinary bladder mucosal polyploidal masses measuring 1.5 × 0.9, 1, and 0.5 cm were observed, highly suspicious for primary urothelial neoplasms.

**FIGURE 3:**
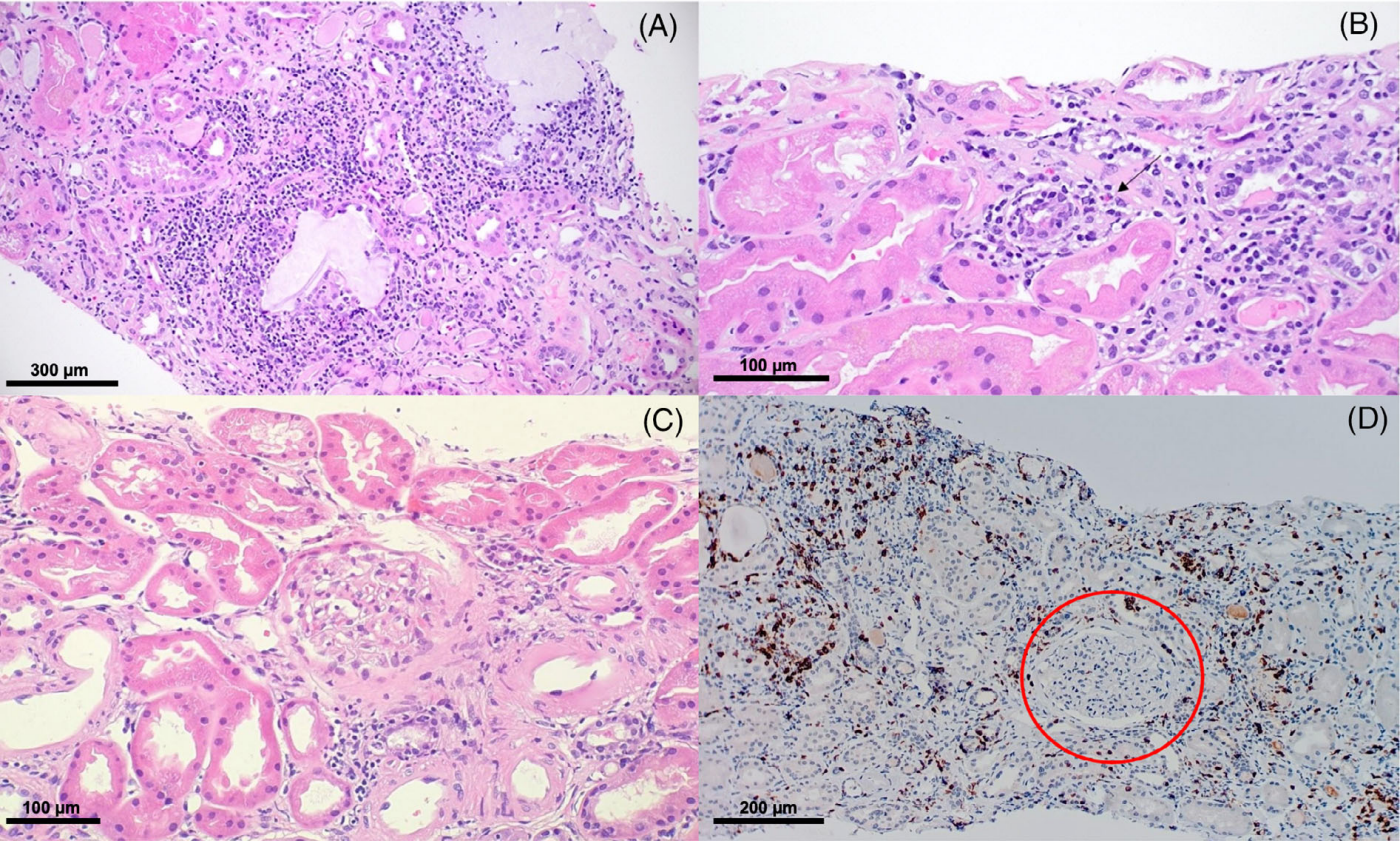
Left kidney biopsy findings showing a mixed inflammatory infiltrate comprised of mature lymphocytes, plasma cells, macrophages, and scattered eosinophils, associated with interstitial edema (A; Hematoxylin & Eosin; 10× magnification). Scattered eosinophils (*arrow*) are visible at greater magnification, in addition to surrounding focal tubulitis (B; Hematoxylin & Eosin; 40×). A single intact glomerulus is observed amidst a background of infiltrating mature lymphocytes (C; Hematoxylin & Eosin; 10×). Immunohistochemical staining highlights infiltrating CD8+ lymphocytes, with a rare glomerulus also present (D; CD8; 20×).

On routine check‐up for cycle five of atezolizumab, the patient was found to have an increased creatinine of 2.2 mg/dL compared to a baseline of 1.3–1.4 mg/dL. He was asymptomatic with normal urinalysis findings and otherwise stable labs. The decision was made to proceed with treatment and close future monitoring of renal function. At the next follow‐up preceding cycle six, the patient mentioned decreased urine output and some dysuria despite good fluid intake. He additionally reported resolved symptoms of fatigue, subjective fever, and chills from ten days prior, likely attributed to later‐confirmed COVID‐19 infection. A basic metabolic panel revealed a serum creatinine of 4.7 mg/dL, and treatment was held due to concern for acute kidney injury (Stage 3 per KDIGO criteria). The patient was subsequently admitted for workup. Lung x‐ray showed no acute changes, and renal ultrasound showed no evidence of acute renal pathology or hydronephrosis; however, a few urinary bladder polypoidal masses suspicious for urothelial neoplasms were incidentally found (Figure 2). Urinalysis and microscopy revealed trace protein, many white blood cells (WBCs), hyaline casts with WBCs, and occasional renal tubular epithelial cells. His calculated fractional excretion of sodium (FENa) was 2.8%. To confirm the diagnosis of possible intrinsic renal injury, an ultrasound‐guided non‐focal biopsy of the left kidney was performed, revealing infiltration of mature lymphocytes, including CD8+ T cells, as well as plasma cells, macrophages, and scattered eosinophils with relatively preserved glomeruli and renal vasculature, overall consistent with AIN (Figure 3). A Naranjo algorithm score of 6 was determined, indicating the nephrotoxicity event was a ‘probable’ adverse drug reaction (see Data S1). Throughout the ten‐day admission, the patient was afebrile with stable vitals on room air and minimal symptomatic complaints.

The patient was discharged on 80 mg of prednisone daily (1 mg/kg), with a taper plan to be determined at outpatient follow‐up. Adjustments were also made to the patient's diabetes medications. Due to kidney injury, metformin and glimepiride were discontinued and replaced by glipizide and sitagliptin, factoring in medication affordability, as well. Given a concern for steroid‐induced hyperglycemia, the dose of glipizide was increased to 5 mg daily. Additionally, his prior‐to‐admission proton‐pump inhibitor (PPI) was discontinued.

At a three‐week follow‐up visit, the patient's creatine had improved to 2.5 mg/dL with no other signs or symptoms of kidney dysfunction. With the continued steroid treatment, he experienced significant fluctuations in blood sugar, averaging around 330 mg/dL. His steroid dose was decreased to 60 mg with plans to further taper based on follow‐up renal labs. Discussion regarding ICI resumption was to occur following completion of the steroid course. Unfortunately, a few weeks later, the patient developed acute hypoxic respiratory failure and was found to have positive serum fungitell and beta‐D‐glucan, as well as diffuse multifocal ground‐glass opacities concerning for *Pneumocystis jiroveci* pneumonia (PJP). He was treated with a 21‐day course of trimethoprim‐sulfamethoxazole. Given the patient's worsening status, he was transitioned to hospice care and passed away shortly after.

## 3 DISCUSSION

Multiple organs are vulnerable to the off‐target effects of ICIs. Insults to the kidney, in particular, are difficult to diagnose clinically, and there is no consensus on optimal management strategies when confronted with uncertain etiologies. In this report, we described a rare case of ICI‐associated AIN in HCC, for which atezolizumab and bevacizumab combination therapy was recently approved, where biopsy elucidated the etiology of suspected AKI. Unlike pre‐renal causes that are often remedied by increasing fluid intake, intrarenal pathologies, like AIN, require prompt immunosuppressive treatment and discontinuation of potential offending agent(s) to maximize chances for complete kidney recovery, as evidenced by prior studies.^3^, ^12^

Clinical features cannot reliably distinguish ICI‐induced AKI from alternative causes; however, suspicion typically arises when elevated sCr is accompanied by sterile pyuria, mild proteinuria, and sometimes microhematuria in the absence of hypertension, edema, uremia, and electrolyte disturbances.^13^ Patients rarely exhibit the classic triad of fever, eosinophilia, and rash, though they may present with at least one preceding or concurrent extra‐renal irAE.^12^ Risk factors for renal irAEs include concomitant NSAID/PPI use, advanced age (>65 years), and lower baseline estimate glomerular filtration rate.^12^ Here, we presented a patient who notably exhibited elevated sCr, sterile pyuria, trace proteinuria, and elevated FENa (>2), raising suspicion for intrinsic pathology.

Kidney biopsy is recognized as the gold standard for establishing an etiology of suspected AKI.^5^ Nevertheless, in practice, the decision for its utilization is complex and subjective. The National Comprehensive Cancer Network recommended considering kidney biopsy only in patients with more than a threefold increase in sCr.^14^ In contrast, the American Society of Clinical Oncology recommends proceeding directly with corticosteroids and monitoring sCr and proteinuria without kidney biopsy if other potential sources of AKI can be excluded on clinical grounds and moderate/severe signs of renal irAE are absent.^15^ This approach is risky as it can inappropriately expose patients with other histologic lesions to glucocorticoids or lead to discontinuation of potentially life‐saving ICI therapy erroneously presumed to be the culprit of AKI.^12^ Regardless, while biopsy allows for a more informed initial management strategy based on severity and type of intrinsic pathology, further review of diagnostic data and treatment outcomes for patients with ICI‐AKI may aid in reaching an updated and standardized consensus.

In the present case, biopsy revealed infiltration of mature lymphocytes, including CD8+ T cells, as well as plasma cells, macrophages, and scattered eosinophils. A case of AIN from atezolizumab‐bevacizumab treatment for RCC demonstrated similar findings, with a diffuse inflammatory infiltrate consisting of both T‐helper and cytotoxic T cells, plasma cells, and eosinophils with preserved glomerular architecture.^9^ These findings are consistent with the mechanism underlying irAEs, which is an excessive T‐cell response mediated by cytokine activation causing an increased presence of cytotoxic lymphocytes and a diffuse inflammatory infiltrate.^2^

Although the patient developed a mild case of COVID‐19 during his treatment course, COVID‐19‐associated kidney injury—rare in itself—tends to demonstrate evidence of tubular damage or necrosis,^16^, ^17^, ^18^ which was not observed histologically here. Furthermore, the timeline of the presented patient's AKI appears to precede infection, suggesting ICI therapy as the more likely primary etiology of AIN and further illustrating histology's role in distinguishing etiologies. Hesitation to biopsy is often attributed to the ill, at‐risk condition of cancer patients who are host to multiple comorbidities. Still, renal biopsy should be considered when feasible, especially in the presence of nonspecific manifestations of competing etiologies of AKI and rising findings reported in the literature of glomerular lesions with ICI therapy.^3^, ^5^, ^12^, ^13^, ^19^

A surveillance study by Chen et al.^6^ investigating renal irAEs across other cancer types found the median onset of atezolizumab‐related kidney injury in 165 cases was 63 days, with a range of 20–167 days, often occurring in male patients over the age of 65. It is therefore beneficial to closely monitor for early signs and symptoms of AKI at all points of ICI treatment and perhaps take precautionary measures earlier on to prevent exacerbating injury. Although a risk for recurrent ICI‐AKI or an additional irAE exists, therapies like atezolizumab‐bevacizumab demonstrate favorable tumor responses with improvements in overall survival and are sometimes the only therapeutic option left for patients, underlying the valuable utility of kidney biopsy in navigating the challenging decision to re‐initiate ICI therapy following AKI.^5^, ^12^

The presence of other irAEs and comorbidities, such as diabetes, are also essential to consider in the development of an optimal treatment strategy. While the described case did not have confirmed evidence of other irAEs, the patient's history of diabetes was taken into account upon prescription of prednisone—adjustments were made to the patient's diabetes treatment and instructions for closer at‐home blood glucose monitoring were provided. Typically, steroid tapers for irAEs occur over 4–6 weeks, however this can vary patient‐to‐patient with a reported median total duration of 84.2 days and range of 3–163 days in a prior study evaluating outcomes in 103 patients receiving steroid treatment for irAEs.^20^ While the patient exhibited an initial partial recovery in kidney function after steroids, he eventually decompensated from an opportunistic infection prior to consideration of ICI rechallenge, underscoring a complex interplay of factors that contribute to mortality risk despite clinical intervention.

## 4 CONCLUSION

Combined atezolizumab‐bevacizumab therapy is relatively new compared to other ICIs, thus data capturing accurate incidence rates of irAEs is still lacking. This paucity is pronounced in the setting of renal injury, as the tendency to forego biopsy leads to an underestimation of specific and confirmed pathologies related to ICI usage. In addition to increased vigilance for irAEs from novel ICI agents, diagnosis with renal biopsy when warranted and prompt treatment are critical for preventing irreversible organ damage and improving chances for full kidney recovery in the setting of AKI.

## AUTHOR CONTRIBUTIONS

**Reema Patel:** Writing – original draft; writing – review and editing; data curation. **Omar Elghawy:** Conceptualization; data curation; writing – review and editing. **Amanda Gibbs:** Data curation. **Srishti Gupta:** Data curation. **Varinder Kaur:** Supervision; writing – review and editing.

## CONFLICT OF INTEREST STATEMENT

The authors have stated explicitly that there are no conflicts of interest in connection with this article.

## ETHICS STATEMENT

This work was reviewed by the University of Virginia Institutional Review Board and was approved according to protocol #HSR 24436. All research was conducted in a HIPAA compliant manner and in accordance with the Declaration of Helsinki. The patient's next of kin provided informed consent for publication of the case report.

## Supporting information


**Data S1.** Supporting Information.

## Data Availability

The data available in this paper can only be reached by the authors. As this is a case report, anonymization is strictly applied.
