# Supplementary material for: A case of biopsy‐proven acute interstitial nephritis following atezolizumab‐bevacizumab treatment of advanced unresectable hepatocellular carcinoma
Source: Cancer Rep (Hoboken). 2024 Jul 25;7(7):e2110. doi: 10.1002/cnr2.2110 (PMC11270322; doi:10.1002/cnr2.2110)
Supplement: Supplementary file 1 — Data S1. Supporting Information. [file CNR2-7-e2110-s001.docx]

Naranjo Algorithm

| Naranjo Criterion |  |
| --- | --- |
| 1.Are there previous conclusive reports on this reaction? | No [0] |
| 2. Did adverse event appear after the suspected drug was given? | Yes [+2] |
| 3. Did the adverse reaction improve when the drug was discontinued, or a specific antagonist was given? | Yes [+1] |
| 4. Did the adverse reaction appear when the drug was readministered? | Do not know or was not done [0] |
| 5. Are there alternative causes that could have caused the reaction? | No [+2] |
| 6. Did the reaction reappear when a placebo was given? | Do not know or was not done [0] |
| 7. Was the drug detected in any body fluid in toxic concentrations? | Do not know or was not done [0] |
| 8. Was the reaction more severe when the dose was increased, or less severe when the dose was decreased? | Do not know or was not done [0] |
| 9. Did the patient have a similar reaction to the same or similar drugs in any previous exposure? | Do not know or was not done [0] |
| 10. Was the adverse event confirmed by any objective evidence? | Yes [+1] |

Naranjo Score: 6

**Probable Adverse Drug Reaction**
